# Which Nonsurgical Treatments Do Patients Believe Are Most Effective for Hip and Knee Arthritis?

**DOI:** 10.5435/JAAOSGlobal-D-20-00046

**Published:** 2020-05-12

**Authors:** Cindy R. Nahhas, Brian C. Fuller, Charles P. Hannon, Tad L. Gerlinger, Denis Nam, Craig J. Della Valle

**Affiliations:** From the Department of Orthopaedic Surgery, Rush University Medical Center, Chicago, IL.

## Abstract

**Background:**

The purpose of this study was to determine which nonsurgical treatments patients believe are most effective for managing pain secondary to hip and knee arthritis.

**Methods:**

Five hundred sixty-five consecutive patients were administered an anonymous questionnaire developed in consultation with a center with expertise in survey design. Statistical analyses included Student *t*-test, Fisher Exact, Wilcoxon Rank-Sum test, and generalized cost-effectiveness analysis.

**Results:**

Four hundred thirty-six patients completed the questionnaire (response rate 77.2%). Opioids (52 of 118; 44.1%), prescription nonsteroidal anti-inflammatory drugs (NSAIDs) (67 of 200; 33.5%), and corticosteroid injections (87 of 260; 33.5%) were reported as most effective. Stem cell and platelet-rich plasma injections were selected by three of 12 (25.0%) and three of 15 patients (19.5%), respectively, and physical therapy (PT) by 50 of 257 patients (19.5%). Twenty-five percent of respondents received opioids, commonly prescribed by primary care providers (48.2%) and orthopaedic surgeons (39.5%). Opioid use correlated with lower patient-reported effectiveness of PT, NSAIDs, and corticosteroid injections (*P* < 0.05). The highest cost-effectiveness ratios were NSAIDs, opioids, and acetaminophen (2.2, 3.7, 4.0, and 5.4, respectively). The lowest cost-effectiveness ratios were stem cell injections, platelet-rich plasma injections, and PT (1966.7, 520.8, and 138.6, respectively).

**Conclusions:**

The nonsurgical treatments that are reported by patients to be most effective are oftentimes the least expensive.

The pain associated with osteoarthritis can have a detrimental impact on a patient's function and quality of life.^1,2^ Total joint arthroplasty is an effective treatment option for end-stage osteoarthritis,^3,4^ but a trial of conservative management is prudent and should be tried before surgical intervention. There are many nonsurgical treatment options available to patients for the treatment of hip and knee osteoarthritis.^2,5,6^

The American Academic of Orthopaedic Surgeons (AAOS) published the guidelines to assist in making evidence-based decisions in the conservative management of hip and knee osteoarthritis.^7,8^ Studies support the use of strengthening, low impact aerobic exercise, weight loss, nonsteroidal anti-inflammatory drugs (NSAIDs), and tramadol in the management of knee osteoarthritis.^1,9,10,11,12^ Physical therapy (PT), NSAIDs, and intraarticular corticosteroids are recommended for conservative treatment of hip osteoarthritis.^13-15^ Inconclusive evidence exists regarding the use of acetaminophen, opioids, and platelet-rich plasma (PRP) injections, and hyaluronic acid injections.^7,8^ In addition, the literature largely consists of studies comparing the effectiveness of one or two treatment options and lacks a study of patients' attitudes toward which conservative treatments are most effective.

Despite AAOS recommendations, many patients undergo conservative treatment modalities that are either not endorsed or have inconclusive evidence.^2,16,17^ A recent study by Bedard et al.^16^ demonstrated that if only AAOS-recommended treatment modalities were used, the cost associated with treatment of osteoarthritis of the knee could be decreased by 45% in the year before arthroplasty. With the contemporary emphasis on minimizing healthcare costs, careful consideration should be given to the cost benefit ratio of conservative treatments for osteoarthritis of the hip and knee. The purpose of this study was to determine which nonsurgical treatments that patients reported are most effective for managing pain secondary to hip and knee arthritis and to determine the cost-effectiveness of each treatment modality.

## Methods

### Study Population and Questionnaire Administration

A consecutive series of 565 new patients presenting to one of three arthroplasty surgeons at a single academic medical center were invited to participate in this study. All patients were undergoing their initial visit for arthroplasty evaluation. All patients must have been diagnosed with arthritis by a physician before being eligible to schedule an appointment with any of the three surgeons. An anonymous questionnaire was given to patients at the start of their first clinical appointment. The survey was administered only once to patients presenting to clinic over a 5-month period from November 1, 2018, to April 1, 2019. The 32-question survey was distributed by a single mode (paper) and was self-administered (Appendix 1, http://links.lww.com/JG9/A75). The voluntary and anonymous nature of the questionnaire was outlined to patients in a cover letter attached to the survey. Patients were given sufficient time to complete the questionnaire in addition to the standard new patient registration forms. The institutional review board granted exemption of this study.

### Questionnaire Design

A survey of 32 questions was created in collaboration with the University of Wisconsin Survey Center.^18,19^ Before commencement of this study, a small sample of patients were asked to partake in cognitive interviewing, a process in which a participant reads the questionnaire aloud and explains their reasoning as they answer questions.^20^ This methodology helped to identify any words or sentences that might have been misunderstood by patients. The survey was revised accordingly to address the concerns raised by cognitive interviewing. The administered questionnaire is available in Appendix 1, http://links.lww.com/JG9/A75.

### Questionnaire Content

The questionnaire asked the respondents to think only about the hip or knee that was causing the most pain at the time they took the survey. The question items inquired about whether a patient had tried a particular nonsurgical treatment as a way to reduce pain in their hip or knee. If they indicated that they had tried a particular treatment, they were then asked how effective it was in reducing their pain on a scale (five point Likert scale: one not at all, two a little, three somewhat, four very, and five extremely).^21^ Some items had additional follow-up questions, including which type of physician prescribed a particular treatment. The nonsurgical treatments survey included PT, acetaminophen, over-the-counter (OTC) NSAIDs, prescription NSAIDs, opioids, corticosteroid injections, hyaluronic acid injections, PRP injections, stem cell injections, and assistive walking devices. Each pharmaceutical treatment type listed examples of generic and brand names to assist respondents in recognizing the pharmaceutical in question.

After step-wise questioning of each treatment type, respondents were then asked to choose the single most effective treatment in reducing the pain in their hip or knee. The final portion of the questionnaire inquired about demographic information including age, joint (hip or knee), sex, race and ethnicity, education level, and insurance type.

### Statistical Analyses

Descriptive statistics were used to provide the raw survey results. Statistical analyses included the Student *t*-test and Fisher exact test for categorical variables and the Wilcoxon Rank-Sum test for continuous variables. A generalized cost-effectiveness analysis^22^ was performed using each recipient's individual rating of effectiveness on the Likert scale, and dollar amounts were derived from the previously published data on cost^16^ and institutional data on the mean cost for treatment event at the site this study was performed. All analyses were performed with Stata 14.2 (Stata Corp, College Station, TX) using alpha = 0.05.

## Results

Four hundred thirty-six patients completed the questionnaire (response rate 77.2%). Respondents were a mean age of 61.6 ± 10.9 years (range, 24 to 90 years old). Respondents were 45.2% men, 75.5% white, and 77.8% had at least some college education. The questionnaire enabled respondents to identify more than one insurance type with Medicare or Medicaid being most common, followed by insurance through an employer (Table 1).

**Table 1:** Respondent Demographics

Demographic	Number (percent)
Age at survey (years)	61.6 ± 10.92
Sex, male	191 (45.2)
Insurance type	
Employer	163 (43.1)
Spouse's employer	76 (20.8)
Veteran's association	5 (1.4)
Purchased directly	45 (12.4)
Medicaid and/or Medicare	171 (44.8)
Other	46 (13)
Racial identity	
White	329 (75.5)
Hispanic or Latino	39 (8.9)
Black or African American	48 (11)
Asian	10 (2.3)
Other	3 (0.7)
American Indian or Alaskan Native	2 (0.5)
Native Hawaiian or Pacific Islander	1 (0.2)
Highest education level	
Some high school	16 (3.9)
High school diploma	59 (14.5)
Trade school	15 (3.7)
Some college	74 (18.2)
Associate's degree	28 (6.9)
Bachelor's degree	106 (26.1)
Master's degree	74 (18.2)
Advanced degree	34 (8.4)

**Figure 1:**
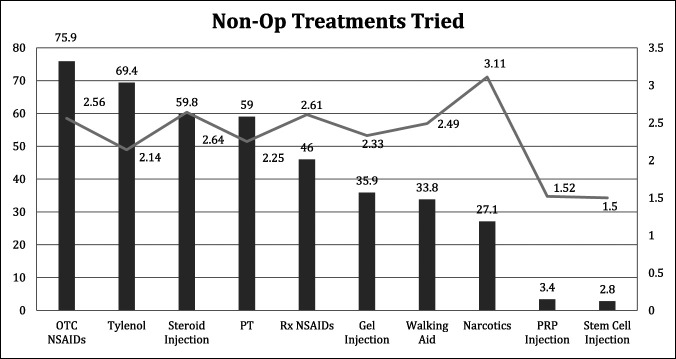
The proportion of survey participants who tried each nonsurgical treatment is represented concurrently with the mean rated effectiveness of that treatment, as rated on a five-point Likert scale. NSAID = nonsteroidal anti-inflammatory drug; OTC = over the counter; PT = physical therapy; PRP = platelet-rich plasma; Rx = prescription.

Before presenting for arthroplasty evaluation, respondents tried a mean of 4.1 ± 1.9 (range, 0 to 9) nonsurgical treatments for their pain. The most commonly used treatments were OTC NSAIDs (75.9%), acetaminophen (69.4%), intra-articular corticosteroid injections (59.8%), and PT (59.0%) (Table 2 and Figure 1). When asked to pick the most effective of treatments of the ones they have tried and adjusting for only the treatments each patient had tried, patients preferred opioids (44.1%), corticosteroid injections (33.5%), and prescription NSAIDs (33.5%). PRP was preferred by three of the 15 patients who received it. Stem cell injections were also preferred by three of 12 patients (25.0%) who tried the treatment. PT was the preferred treatment of 50 of 257 patients who received PT (19.5%) (Table 3). No differences were observed between hip and knee patients.

**Table 2:** Proportion of Respondents Who Tried Each Treatment and Corresponding Rated Effectiveness^a^

Treatment	Number (percent) (n = 435)	Mean Reported Effectiveness
Over-the-counter NSAIDs	330 (75.9%)	2.56 ± 0.91
Acetaminophen	302 (69.4%)	2.14 ± 0.90
Corticosteroid injection	260 (59.8%)	2.64 ± 1.20
Physical therapy	257 (59.0%)	2.25 ± 0.98
Prescription NSAIDs	200 (46.0%)	2.61 ± 1.00
Gel injection	156 (35.9%)	2.33 ± 1.19
Assistive walking device	147 (33.8%)	2.49 ± 0.91
Opioids	118 (27.1%)	3.11 ± 1.11
Platelet-rich plasma injection	15 (3.4%)	1.52 ± 1.04
Stem cell injection	12 (2.8%)	1.50 ± 1.00

NSAID = nonsteroidal anti-inflammatory drug; PT = physical therapy

a As rated on a five point Likert scale

**Table 3:** Nonsurgical Treatment Selected as Most Effective

Nonsurgical Treatment	Number Selected (Percent of Total)	Percent, Adjusted for Respondents Who Used the Treatment	Mean CER (SD)
Opioids	52 (11.9%)	44.1%	4.0 (7.3)
Corticosteroid injection	87 (20.0%)	33.5%	22.7 (31.4)
Prescription NSAID	67 (15.4%)	33.5%	3.7 (5.6)
Gel injection	41 (9.4%)	26.3%	104.0 (122.7)
Stem cell injection	3 (0.7%)	25.0%	1966.7 (1428.1)
Over-the-counter NSAID	77 (17.7%)	23.3%	2.2 (3.4)
Physical therapy	50 (11.5%)	19.5%	138.6 (166.1)
Platelet-rich plasma injection	3 (0.7%)	19.5%	520.8 (333.9)
Assistive walking device	22 (5.0%)	15.0%	9.2 (13.3)
Acetaminophen	32 (7.3%)	10.6%	5.4 (6.3)

CER = Cost-effectiveness Ratio; NSAID = nonsteroidal anti-inflammatory drug

The most cost-effective treatments were OTC and prescription NSAIDs, narcotics, and acetaminophen (mean cost-effectiveness ratio were 2.2, 3.7, 4.0, and 5.4, respectively). The least cost-effective treatment by far was stem cell injections, followed by PRP injections and PT (mean cost-effectiveness ratios were 1966.7, 520.8, and 138.6, respectively) (Table 3).

Twenty-seven percent of respondents received opioids, and these patients were significantly younger and less educated compared with patients who did not try them (Table 4). Respondents who tried opioids as a nonsurgical treatment had significantly lower patient-reported effectiveness of other treatment modalities, including PT, acetaminophen, corticosteroid injections, and gel injections, compared with patients who did not try opioids (*P* < 0.05 for all) (Table 4). These were most commonly prescribed by primary care providers (48.2%) and orthopaedic surgeons (39.5%) (Table 5).

**Table 4:** Reported Effectiveness^a^ of Nonsurgical Treatments Compared Among Patients Who Did and Did Not try Opioids

Demographic	Tried Narcotic	*P*	Did Not Try Narcotic
Age (years)	58.25 ± 11.7	<0.001^b^	62.88 ± 10.42
College educated	28 (24.2%)	0.047^c^	108 (34.4%)

Treatment (Percent That Tried Both Opioids and Treatment Listed)	Tried Narcotic	*P*	Did Not Try Narcotic
Physical therapy (69.8% tried)	1.94 ± 0.87	<0.001^d^	2.40 ± 1.01
Acetaminophen (77.6% tried)	1.75 ± 0.85	<0.001^d^	2.31 ± 0.88
Over-the-counter NSAID (83.6% tried)	2.19 ± 0.89	<0.001^d^	2.71 ± 0.89
Prescription NSAID (63.8% tried)	2.41 ± 1.06	0.028^d^	2.74 ± 0.94
Corticosteroid injection (74.1% tried)	2.42 ± 1.17	0.036^d^	2.76 ± 1.21
Hyaluronic acid injections (47.4% tried)	2.06 ± 1.12	0.039^d^	2.48 ± 1.20
Platelet-rich plasma injection (9.5% tried)	1.18 ± 0.60	0.094^d^	1.83 ± 1.27
Stem cell injection (3.4% tried)	1.75 ± 1.50	0.824^d^	1.38 ± 0.74
Assistive walking device (49.1% tried)	2.42 ± 0.92	0.535^d^	2.53 ± 0.92

NSAID = nonsteroidal anti-inflammatory drug; PT = physical therapy.

a As rated on a five point Likert scale.

b Student *t*-test.

c Fisher Exact.

d Wilcoxon Rank-Sum test, used when a Student *t*-test was invalid because of the departures from normality.

**Table 5:** Opioid Prescribing Doctor

Physician Type	Total Reported (Percent^a^)
Primary care doctor (family medicine or internal medicine doctor)	55 (48.2%)
Orthopaedic surgeon	45 (39.5%)
Pain management specialist	21 (18.4%)
Other type of doctor (emergency medicine or leftover from previous surgery)	10 (8.8%)
Rheumatologist	7 (6.1%)
Primary care sports medicine doctor	3 (2.6%)

a Percent totals to more than 100% because patients could report more than one prescribing doctor. One hundred fourteen patients answered this question when surveyed and percentages are of total.

## Discussion

The results from this study demonstrated that opioids, intra-articular corticosteroid injections, and NSAIDs are reported by patients as the most effective nonsurgical modalities for reducing pain from hip and knee arthritis. These three treatments were also among the most cost-effective treatments surveyed. PRP injections, stem cell injections, and PT were reported by patients to be the least effective treatments for reducing pain and were the least cost-effective treatments surveyed.

These findings are reassuring because the use of NSAIDs is supported by the AAOS Clinical Practice Guidelines for both the hip and knee, whereas the use of intra-articular corticosteroids for the hip is also supported.^7,8^ It is well established that NSAIDs improve pain and function for the treatment of osteoarthritis and are generally considered safe and effective drugs.^12,23^ Before recommending NSAIDs physicians should assess the patients' comorbidities and tolerance to these medications because there is potential for adverse effects including renal toxicity and peptic ulcers.^12^ Similarly, intra-articular corticosteroid injections have demonstrated improvement in pain and function in osteoarthritis knees^24,25^ and hips.^13-15^

Opioids were used by 27% of respondents and were considered highly effective by patients in this study. AAOS Clinical Practice Guidelines are inconclusive regarding opioid use as a conservative treatment of arthritis; however, more recent evidence suggests that the risks may outweigh the benefits. Opioids are often prescribed to patients who are either intolerant to or have failed other pharmacologic options. Although a significant portion of patients preferred opioids in our study, a systematic review of randomized controlled trials by da Costa et al.^26^ found that opioids provide a small clinical benefit but are associated with significant adverse events. Opioids also pose significant risks to patients and their families because of the risk of diversion and addiction. There are also specific risks of opioid use and arthroplasty with strong evidence suggesting that patients on preoperative opioids do poorly after arthroplasty compared with opioid naïve patients.^27-29^ Patients using opioids before total joint arthroplasty have worse outcomes after surgery, including less pain relief and greater risk of periprosthetic joint infection.^29-31^ Interestingly, our study found that patients who tried opioids as a conservative treatment also had significantly lower rated effectiveness of other treatments including NSAIDs, corticosteroid injections, and PT. Although no conclusions can be drawn regarding causation or temporality, it is concerning that having tried opioids correlates with lower effectiveness of other treatment modalities. The American Association of Hip and Knee Surgeons' position is that opioids should only be used to treat osteoarthritis in exceptional circumstances.^32^ Patient and physician education regarding the risks associated with opioids for the treatment of arthritis is necessary to help mitigate adverse effects and improve outcomes after total joint arthroplasty (TJA).

Intra-articular PRP injections and stem cell injections were not commonly used by respondents in our study and had the lowest rated clinical efficacies of all treatments surveyed. The AAOS Clinical Practice Guidelines report inconclusive evidence on the use of PRP injections and have no statement on stem cell injections. Current evidence on the clinical effectiveness of PRP for treatment of osteoarthritis is inconclusive.^33^ Most studies have compared PRP with hyaluronic acid, with some demonstrating superiority of PRP^34,35^ with others concluding no difference.^36,37^ Hyaluronic acid itself is not recommended by the AAOS Clinical Practice Guidelines, and thus, even if PRP has shown superiority over hyaluronic acid, it is difficult to draw conclusions regarding whether it should be used in this context. In addition, there is a paucity of level I evidence on the use of stem cell injections for the treatment of osteoarthritis. The few studies published on stem cells for arthritis demonstrated favorable outcomes, but their the methodology has been questioned.^38^ In addition to the questionable clinical effectiveness of PRP and stem cells, there are safety and cost concerns with these treatments. Although both treatments are generally considered to be safe, any injection into the knee poses a risk for infection to patients. Both PRP and stem cell treatments are also not covered by most commercial insurances including Medicare leading to significant out of pocket expenses for patients. Given these concerns, the American Association of Hip and Knee Surgeons (AAHKS) released a position statement in June 2019 recommending against the use of PRP and stem cells for treatment of advanced hip or knee arthritis.^39^ Further prospective randomized controlled trials examining both the clinical and cost efficacy of these treatments are necessary before they should be routinely used for the conservative treatment of hip and knee osteoarthritis.

With increases in healthcare utilization and subsequently healthcare costs, a critical evaluation of how healthcare dollars are spent is necessary. Innovative new alternative payment models, such as bundle payments, have been developed to emphasize value, defined as the ratio of the clinical benefit of a treatment over the costs associated with that treatment, when making treatment decisions.^40^ Bundling of payments has to date been at the procedure level, but recent models have bundled payments for treatment of diseases, such as arthritis, to encourage value-based care throughout the care pathway. A recent study reported that the outpatient cost of knee osteoarthritis in the year before total knee arthroplasty was over $43 million.^16^ Our study found that AAOS-recommended treatment modalities, namely NSAIDs and intra-articular corticosteroid injections, were preferred by patients and were also of the most cost-effective treatments. Stem cell injections had the lowest rated clinical efficacy in our study and were by far the least cost-effective treatment. Similarly, hyaluronic acid injections are not recommended by the AAOS Clinical Practice Guidelines, yet it has been found that these injections alone account for 29% of the cost of nonsurgical treatment of the knee in the year before total knee arthroplasty.^16^ The value of nonsurgical treatment modalities in combination with AAOS Clinical Practice Guidelines should be considered when choosing a treatment plan for patients with symptomatic arthritis. The results from our study suggest that for conservative treatment of arthritis NSAIDs and intra-articular steroid injections are of the most clinical efficacious treatments and also most cost-effective. These treatments should be the first-line treatments for conservative management of arthritis.

Several limitations should be considered when interpreting the results from this study. First, all survey participants were presenting to an arthroplasty clinic and thus had likely already failed conservative treatment of their arthritis. This may result in recall bias. In addition, there may be selection bias associated with our response rate of 77.2%. Third, the study was performed at a single academic institution, and the respondents were relatively homogenous. Most patients identified as Caucasian with at least some college education and the results should be considered in this context. Furthermore, because of the anonymous nature of the study, the severity of arthritis for each respondent cannot be assessed, and conclusions cannot be drawn regarding which treatment may be superior at different stages of disease severity. Our study focuses on treatment modalities in isolation, but patients may have used multiple treatment modalities concomitantly and we were not able to control for this occurrence. Finally, the questionnaire item regarding the single most effective treatment emphasized that patients should choose one and only one treatment, but many respondents failed to select an answer, whereas others chose more than one response. Each chosen response was considered in our results, as to not exclude patients who did not fully follow the instructions.

Intra-articular corticosteroids, NSAIDs, and opioids were reported to be the most effective and were among the most cost-effective treatments surveyed. PRP, stem cells, and PT were the least effective and were the least cost-effective treatments surveyed. Clinical practice guidelines support the use of exercise, weight loss, and anti-inflammatory drugs in the knee and exercise, anti-inflammatory drugs, and corticosteroid injections in the hip. A strikingly high number of patients received opioids for nonsurgical treatment, many given by orthopaedic surgeons. Given the deleterious effects of opioids on perioperative outcomes and the decreased efficacy of other nonsurgical treatments once opioids were given, greater education is needed to discourage their use preoperatively.

## References

[1] Focht BC: Effectiveness of exercise interventions in reducing pain symptoms among older adults with knee osteoarthritis: A review. J Aging Phys Act 2006;14:212-235. 19462551 10.1123/japa.14.2.212

[2] Wood AM Brock TM Heil K Holmes R Weusten A: A review on the management of hip and knee osteoarthritis. Int J Chronic Dis 2013;2013:845015. 26464847 10.1155/2013/845015 PMC4590943

[3] Martinez-Cano JP Herrera-Escobar JP Arango Gutierrez AS Sanchez Vergel A Martinez-Rondanelli A: Prospective quality of life assessment after hip and knee arthroplasty: Short- and mid-term follow-up results. Arthroplast Today 2017;3:125-130. 28695185 10.1016/j.artd.2016.09.008 PMC5485233

[4] Liebs TR Herzberg W Rüther W Russlies M Hassenpflug J: Multicenter arthroplasty aftercare project MAAP. Quality-adjusted life years gained by hip and knee replacement surgery and its aftercare. Arch Phys Med Rehabil 2016;97:691-700. 26792619 10.1016/j.apmr.2015.12.021

[5] Ponnusamy KE Vasarhelyi EM McCalden RW Somerville LE Marsh JD: Cost-effectiveness of total hip arthroplasty versus nonoperative management in normal, overweight, obese, severely obese, morbidly obese, and super obese patients: A Markov Model. J Arthroplasty 2018;33:3629-3636. 30266324 10.1016/j.arth.2018.08.023

[6] Ponnusamy KE Vasarhelyi EM Somerville L McCalden RW Marsh JD: Cost-effectiveness of total knee arthroplasty vs nonoperative management in normal, overweight, obese, severely obese, morbidly obese, and super-obese patients: A Markov Model. J Arthroplasty 2018;33:S32-S38. 29550168 10.1016/j.arth.2018.02.031

[7] Jevsevar DS Brown GA Jones DL: The American Academy of Orthopaedic Surgeons evidence-based guideline on: Treatment of osteoarthritis of the knee, 2nd edition. J Bone Joint Surg Am 2013;95:1885-1886. 24288804 10.2106/00004623-201310160-00010

[8] Management of osteoarthritis of the hip evidence-based clinical practice guideline. Am Acad Orthopaedic Surgeons 2017;26:e434-e436. 10.5435/JAAOS-D-18-00351 30134309

[9] Jamtvedt G Dahm KT Christie A: Physical therapy interventions for patients with osteoarthritis of the knee: An overview of systematic reviews. Phys Ther 2008;88:123-136. 17986496 10.2522/ptj.20070043

[10] Hughes SL Seymour RB Campbell RT: Long-term impact of fit and strong! On older adults with osteoarthritis. Gerontologist 2006;46:801-814. 17169935 10.1093/geront/46.6.801

[11] Juhl C Christensen R Roos EM Zhang W Lund H: Impact of exercise type and dose on pain and disability in knee osteoarthritis: A systematic review and meta-regression analysis of randomized controlled trials. Arthritis Rheumatol 2014;66:622-636. 24574223 10.1002/art.38290

[12] Richette P Latourte A Frazier A: Safety and efficacy of paracetamol and NSAIDs in osteoarthritis: Which drug to recommend? Expert Opin Drug Saf 2015;14:1259-1268. 26134750 10.1517/14740338.2015.1056776

[13] Kullenberg B Runesson R Tuvhag R Olsson C Resch S: Intraarticular corticosteroid injection: Pain relief in osteoarthritis of the hip? J Rheumatol 2004;31:2265-2268. 15517641

[14] Lambert RG Hutchings EJ Grace MG Jhangri GS Conner-Spady B Maksymowych WP: Steroid injection for osteoarthritis of the hip: A randomized, double-blind, placebo-controlled trial. Arthritis Rheum 2007;56:2278-2287. 17599747 10.1002/art.22739

[15] Robinson P Keenan AM Conaghan PG: Clinical effectiveness and dose response of image-guided intra-articular corticosteroid injection for hip osteoarthritis. Rheumatology (Oxford) 2007;46:285-291. 16873380 10.1093/rheumatology/kel217

[16] Bedard NA Dowdle SB Anthony CA: The AAHKS clinical research award: What are the costs of knee osteoarthritis in the year prior to total knee arthroplasty? J Arthroplasty 2017;32:S8-S10.e11. 28209276 10.1016/j.arth.2017.01.011

[17] Dhawan A Mather RC Karas V: An epidemiologic analysis of clinical practice guidelines for non-arthroplasty treatment of osteoarthritis of the knee. Arthroscopy 2014;30:65-71. 24290788 10.1016/j.arthro.2013.09.002

[18] (CDC) CfDCaP: Prevalence of cholesterol screening and high blood cholesterol among adults—United States, 2005, 2007, and 2009. MMWR Morb Mortal Wkly Rep 2012;61:697-702. 22951451

[19] Radler BT Ryff CD: Who participates? Accounting for longitudinal retention in the MIDUS national study of health and well-being. J Aging Health 2010;22:307-331. 20103686 10.1177/0898264309358617 PMC2837791

[20] Willis GB Artino AR: What do our respondents think we're asking? Using cognitive interviewing to improve medical education surveys. J Grad Med Educ 2013;5:353-356. 24404294 10.4300/JGME-D-13-00154.1 PMC3771159

[21] Norman G: Likert scales, levels of measurement and the “laws” of statistics. Adv Health Sci Educ Theor Pract 2010;15:625-632. 10.1007/s10459-010-9222-y 20146096

[22] WHO Guide to Cost-Effectiveness Analysis. World Health Organization; 2003.

[23] Bannuru RR Schmid CH Kent DM Vaysbrot EE Wong JB McAlindon TE: Comparative effectiveness of pharmacologic interventions for knee osteoarthritis: A systematic review and network meta-analysis. Ann Intern Med 2015;162:46-54. 25560713 10.7326/M14-1231

[24] Jüni P Hari R Rutjes AW: Intra-articular corticosteroid for knee osteoarthritis. Cochrane Database Syst Rev 2015;10:CD005328. 10.1002/14651858.CD005328.pub3 PMC8884338 26490760

[25] de Campos GC Rezende MU Pailo AF Frucchi R Camargo OP: Adding triamcinolone improves viscosupplementation: A randomized clinical trial. Clin Orthop Relat Res 2013;471:613-620. 23100188 10.1007/s11999-012-2659-y PMC3549166

[26] da Costa BR Nüesch E Kasteler R: Oral or transdermal opioids for osteoarthritis of the knee or hip. Cochrane Database Syst Rev 2014:CD003115. 25229835 10.1002/14651858.CD003115.pub4 PMC10993204

[27] Ivers N Dhalla IA Allan GM: Opioids for osteoarthritis pain: Benefits and risks. Can Fam Physician 2012;58:e708. 23242901 PMC3520677

[28] Howes F Buchbinder R Winzenberg TB: Opioids for osteoarthritis? Weighing benefits and risks: A cochrane musculoskeletal group review. J Fam Pract 2011;60:206-212. 21472151

[29] Smith SR Bido J Collins JE Yang H Katz JN Losina E: Impact of preoperative opioid use on total knee arthroplasty outcomes. J Bone Joint Surg Am 2017;99:803-808. 28509820 10.2106/JBJS.16.01200 PMC5426402

[30] Bell KL Shohat N Goswami K Tan TL Kalbian I Parvizi J: Preoperative opioids increase the risk of periprosthetic joint infection after total joint arthroplasty. J Arthroplasty 2018;33:3246-3251.e3241. 30054211 10.1016/j.arth.2018.05.027

[31] Nguyen LC Sing DC Bozic KJ: Preoperative reduction of opioid use before total joint arthroplasty. J Arthroplasty 2016;31(9 suppl):282-287. 27105557 10.1016/j.arth.2016.01.068

[32] Opioid Use for the Treatment of Osteoarthritis of the Hip and Knee. In: Position of the American Association of Hip and Knee Surgeons: American Association of Hip and Knee Surgeons; 2019.

[33] Bennell KL Hunter DJ Paterson KL: Platelet-rich plasma for the management of hip and knee osteoarthritis. Curr Rheumatol Rep 2017;19:24. 28386761 10.1007/s11926-017-0652-x

[34] Cerza F Carnì S Carcangiu A: Comparison between hyaluronic acid and platelet-rich plasma, intra-articular infiltration in the treatment of gonarthrosis. Am J Sports Med 2012;40:2822-2827. 23104611 10.1177/0363546512461902

[35] Raeissadat SA Rayegani SM Hassanabadi H: Knee osteoarthritis injection choices: Platelet- rich plasma (PRP) versus hyaluronic acid (a one-year randomized clinical trial). Clin Med Insights Arthritis Musculoskelet Disord 2015;8:1-8. 25624776 10.4137/CMAMD.S17894 PMC4287055

[36] Filardo G Kon E Di Martino A: Platelet-rich plasma vs hyaluronic acid to treat knee degenerative pathology: Study design and preliminary results of a randomized controlled trial. BMC Musculoskelet Disord 2012;13:229. 23176112 10.1186/1471-2474-13-229 PMC3532098

[37] Filardo G Di Matteo B Di Martino A: Platelet-rich plasma intra-articular knee injections show No superiority versus viscosupplementation: A randomized controlled trial. Am J Sports Med 2015;43:1575-1582. 25952818 10.1177/0363546515582027

[38] Pas HI Winters M Haisma HJ Koenis MJ Tol JL Moen MH: Stem cell injections in knee osteoarthritis: A systematic review of the literature. Br J Sports Med 2017;51:1125-1133. 28258177 10.1136/bjsports-2016-096793

[39] Browne JA Nho SJ Goodman SB Della Valle CJ: American association of hip and knee surgeons, hip society, and knee society position statement on biologics for advanced hip and knee arthritis. J Arthroplasty 2019;34:1051-1052. 31005436 10.1016/j.arth.2019.03.068

[40] Bozic KJ: Value-based healthcare and orthopaedic surgery. Clin Orthop Relat Res 2012;470:1004-1005. 22302657 10.1007/s11999-012-2267-x PMC3293948

